# Avoiding Intubation in Severe Acute Respiratory Distress Syndrome Due to Mixed Amiodarone Toxicity and Haemophilus Influenzae: A Case of Permissive Hypoxemia

**DOI:** 10.7759/cureus.98563

**Published:** 2025-12-06

**Authors:** Ubaldo La Brocca, Giulia Dina Giuseppina Benincasa, Massimiliano Parlanti Garbero, Michele Grio

**Affiliations:** 1 Department of Anesthesia and Intensive Care, Rivoli Hospital, Rivoli, ITA; 2 Department of Respiratory Medicine, San Luigi Gonzaga University Hospital, Orbassano, ITA

**Keywords:** acute respiratory distress syndrome [ards], aipt(amiodarone-induced pulmonary toxicity), drug-induced lung disease (dild), haemophilus influenzae, high-flow nasal cannula (hfnc), permissive hypoxemia, rox index

## Abstract

Amiodarone pulmonary toxicity (APT) is a diagnostic challenge that can mimic or complicate community-acquired pneumonia. In cases of severe acute respiratory distress syndrome (ARDS), invasive mechanical ventilation is the standard of care but carries significant risks. We report a case of severe ARDS successfully managed without intubation through a strategy of "permissive hypoxemia" and high-dose corticosteroids. A 78-year-old female was admitted to the ICU for acute hypoxic respiratory failure. Initial workup identified *Haemophilus influenzae* on bronchoalveolar lavage, leading to a diagnosis of severe pneumonia. Despite targeted antibiotic therapy, hypoxemia worsened. On day two, a review of historical imaging and the patient's long-term amiodarone therapy raised the suspicion of underlying APT. The steroid regimen was escalated to prednisone 160 mg/day. On day three, the patient reached a nadir PaO2/FiO2 ratio of 58 while on a high-flow nasal cannula at 60 L/min and 100% fraction of inspired oxygen. Despite profound hypoxemia, the patient remained hemodynamically stable. A decision was made to tolerate hypoxemia primarily to avoid ventilator-associated pneumonia, given the high susceptibility to infection induced by high-dose steroids. This strategy relied on the hypothesis of rapid reversibility of the inflammatory toxicity upon steroid escalation. Clinical improvement began on day five. A follow-up CT scan on day 10 demonstrated significant radiological clearance. The patient was discharged without requiring intubation. This case highlights the importance of reviewing historical imaging to identify drug-induced toxicity and suggests that, in selected patients with preserved respiratory drive, a noninvasive approach tolerating severe hypoxemia may be a viable alternative to intubation.

## Introduction

Amiodarone is a widely used antiarrhythmic drug, but its utility is limited by potential life-threatening adverse effects, most notably amiodarone pulmonary toxicity (APT), which affects up to 5-10% of long-term users [1]. Diagnosing APT in the acute setting is notoriously difficult, as its clinical presentation, dyspnea, fever, and pulmonary infiltrates often mimic community-acquired pneumonia (CAP) or heart failure. This diagnostic uncertainty is compounded when an infectious pathogen is co-identified, creating a "double hit" scenario that can mask the underlying toxicity.

Furthermore, the management of severe acute respiratory distress syndrome (ARDS), defined as a PaO2/FiO2 ratio < 100 mmHg, typically mandates invasive mechanical ventilation (IMV). However, recent evidence suggests that in selected phenotypes, spontaneous breathing with high-flow nasal cannula (HFNC) or non-invasive ventilation may be feasible [2]. We present a case of severe ARDS (PiO2/FiO2 ratio nadir 58) triggered by *Haemophilus influenzae* superimposed on chronic APT, successfully managed with high-dose corticosteroids and a strategy of "permissive hypoxemia" without intubation.

## Case presentation

A 78-year-old female was admitted to the intensive care unit (ICU) for acute hypoxic respiratory failure. Her past medical history was significant for valvular heart disease (mechanical mitral valve replacement and tricuspid valvuloplasty) and sick sinus syndrome managed with a dual-chamber pacemaker. She had no history of smoking. Crucially, the patient had a history of paroxysmal atrial fibrillation managed with amiodarone (200 mg, five days/week) for approximately 10 years, resulting in massive cumulative exposure.

She presented to the emergency department with worsening dyspnea. An initial chest X-ray showed bilateral opacities, leading to a diagnosis of severe CAP. Upon ICU admission (day zero), she was tachypneic and hypoxemic (PaO2/FiO2 ~80). Given her cardiac history, cardiogenic edema was considered, but hemodynamic stability, bedside echocardiography, and a normal B-type natriuretic peptide (BNP) level suggested a primary pulmonary etiology.

Laboratory investigations on admission revealed leukocytosis (white blood cell count 10,900/μL with 86% neutrophils), significantly elevated C-reactive protein (CRP) at 20.4 mg/dL (reference range <0.5 mg/dL), and procalcitonin (PCT) at 0.8 ng/mL (reference range <0.5 ng/mL).

On admission, the patient was started on a high-flow nasal cannula (HFNC) with a fraction of inspired oxygen (FiO2) of 0.8. A bronchoalveolar lavage (BAL) with FilmArray (bioMérieux, Marcy-l'Étoile, France) identified *Haemophilus influenzae* (10^6 copies/mL). Following the BAL, a chest computed tomography (CT) scan was performed, confirming diffuse bilateral ground-glass opacities and dense consolidations. Based on the FilmArray result, later confirmed by culture, therapy was optimized with targeted ceftriaxone (2 g twice daily) and standard dexamethasone (8 mg/day).

Despite targeted antibiotics, the patient’s respiratory status deteriorated. On day two, a re-evaluation of the admission CT scan and comparison with a CT scan performed 20 months prior revealed pre-existing basal ground-glass opacities and reticular abnormalities, consistent with chronic drug toxicity. Suspecting an acute exacerbation of APT triggered by the infection, the steroid regimen was escalated to prednisone 80 mg twice daily (160 mg/day).

**Figure 1:**
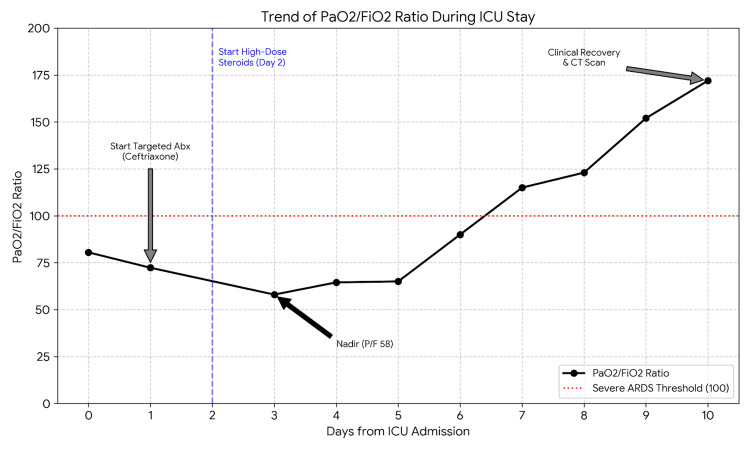
Trend of PaO2/FiO2 ratio during the first 10 days of ICU stay. Note the nadir on day three (PaO2/FiO2 58) followed by rapid recovery after the initiation of high-dose prednisone.

The clinical condition reached its nadir on the morning of day three, with a PaO2/FiO2 ratio of 58 (Figure 1).

The patient was supported solely with HFNC (60 L/min, FiO2 1.0), maintaining an oxygen saturation (SpO2) of approximately 85%. Despite critical hypoxemia, she remained alert, cooperative, and hemodynamically stable (mean arterial pressure > 65 mmHg, heart rate 70 bpm), with no signs of respiratory muscle exhaustion. A decision was made to delay intubation and adopt a strategy of permissive hypoxemia.

From day five, the P/F ratio showed a progressive upward trend. Inflammatory biomarkers significantly improved by day 10, with CRP dropping to 1.1 mg/dL and PCT to 0.07 ng/mL, although a likely steroid-induced leukocytosis persisted (WBC 13,200/μL). 

**Figure 2:**
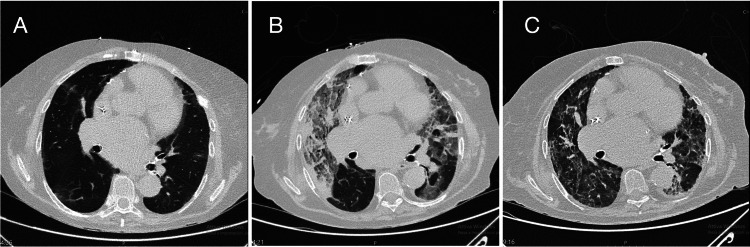
Evolution of non-enhanced axial CT scans at the lung bases. (A) Baseline scan (20 months prior) showing chronic interstitial changes. (B) Admission scan confirming diffuse ground-glass opacities and consolidation. (C) Day 10 scan showing significant radiological clearance.

A follow-up CT scan on day 10 showed dramatic clearance of the consolidations (Figure 2), revealing the underlying interstitial pattern.

The patient was successfully weaned from HFNC to low-flow oxygen and was discharged from the ICU to the Pulmonology Unit on day 15, requiring only minimal oxygen supplementation.

## Discussion

The diagnosis of APT is often one of exclusion. In our case, the isolation of *H. influenzae* acted as a confounding factor. The turning point was the review of historical imaging, which shifted the reasoning from purely infectious ARDS to a mixed etiology. The rapid radiological clearance (10 days) following high-dose steroids confirms the presence of a predominant inflammatory, cortico-sensitive component typical of APT (organizing pneumonia pattern), rather than fixed fibrosis or pure bacterial consolidation.

Managing a patient with a PaO2/FiO2 of 58 without intubation is controversial and contradicts standard prognostic models. Calculating the ROX index (ratio of SpO2/FiO2 to respiratory rate) at the nadir, using the P/F ratio as a surrogate for SpO2/FiO2 due to unreliable oximetry in severe hypoxemia, yielded a value of approximately 2.32 (58/25). An ROX index < 2.85 is traditionally considered a strong predictor of HFNC failure and an indication for immediate intubation [3]. However, our patient exhibited a clear dissociation between these alarming indices and her preserved clinical comfort ("happy hypoxemia") [4]. By overriding the numerical indication for intubation, we prevented the known complications of mechanical ventilation (VAP, barotrauma, hemodynamic collapse) in a fragile patient.

Similar patterns of well-tolerated severe hypoxemia have been extensively described during the COVID-19 pandemic. However, in the broader ARDS population, the prognosis of permissive hypoxemia is not universally favorable. Observational studies have consistently shown that "delayed intubation" after failing non-invasive support is associated with increased mortality compared to early intubation. Therefore, this strategy should not be generalized but strictly reserved for selected patients with rapidly reversible etiologies and close monitoring to avoid the perils of delayed intervention.

A major concern regarding permissive hypoxemia is the potential for long-term neurocognitive sequelae. While recent large-scale trials have suggested that lower oxygenation targets do not increase mortality [5], the impact on long-term cognitive function remains a valid concern. In our case, neurological monitoring was a cornerstone of the noninvasive strategy. The patient maintained a Glasgow Coma Scale (GCS) of 15 throughout the ICU stay, remaining fully alert, cooperative, and oriented even during the nadir of hypoxemia. At the time of discharge, no gross neurological or cognitive deficits were observed, supporting the safety of this approach in this specific patient context.

## Conclusions

This case underscores two key lessons: 1) in patients on chronic amiodarone, always suspect toxicity even when an infection is found; and 2) treating the patient rather than the numbers is feasible. Even with an ROX index predicting failure, a trial of noninvasive support with high-dose steroids may avoid intubation in hemodynamically stable patients.
